# Clinical, neuroimaging, biochemical, and genetic features in six Chinese patients with Adrenomyeloneuropathy

**DOI:** 10.1186/s12883-019-1449-5

**Published:** 2019-09-16

**Authors:** Jie Li, Hongfen Wang, Zizi He, Xiangqing Wang, Jing Tang, Dehui Huang

**Affiliations:** 1 Department of Neurology, Chinese PLA General Hospital, Fuxing Road 28, Haidian District, Beijing, 100853 People’s Republic of China; 2 Department of Radiology, Chinese PLA General Hospital, Beijing, 100853 People’s Republic of China

**Keywords:** Adrenomyeloneuropathy, Addison disease, Spastic paraparesis, Very long chain fatty acid, ABCD1

## Abstract

**Background:**

Adrenoleukodystrophy is a rare neurogenetic disease, AMN is the most common adult phenotype, such patients in China have not gotten enough attention. This article aims to study the features of AMN in Chinese patients and expand the gene spectrum of Chinese X-linked adrenoleukodystrophy (X-ALD) patients.

**Methods:**

We applied clinical analysis, radiology, plasma levels of very long chain fatty acids (VLCFA) and genetic analysis to test the 6 Chinese AMN patients.

**Results:**

All 6 patients are men. Ages of neurological symptom onset are distributed between 21 and 38. Sexual dysfunction occurred in 5 of 6 patients. Three patients had positive family history. Five patients had Addison’s disease. Four patients were diagnosed as pure AMN, while the other two patients were with cerebral involvement. Four patients had abnormalities of nerve conduction studies. There were four patients with central conduction defects in somatosensory evoked potential tests. All 6 patients were found diffuse cord atrophy in spinal MRI. Brain MRI showed abnormal signals in 2 of the 6 tested patients, which indicated the clinical phenotypes. Plasma levels of VLCFA, as well as C24:0/C22:0 and C26:0/C22:0 ratios were elevated in 5 tested patients. Five different ABCD1 mutations were identified in 5 tested patients, one of which was a de novo mutation, and the other four have been reported previously.

**Conclusion:**

This research described the clinical, neuroimaging, biochemical, and genetic sides of Chinese AMN patients. A de novo mutation in the ABCD1 gene sequence was identified. Emotional trauma may trigger or aggravate the development of cerebral demyelination in AMN patients. Regular evaluation of brain MRI is important for AMN patients, especially for ‘pure AMN’ patients. When encountering patients with ‘myeloneuropathy-only’, neurologists should not ignore the tests of VLCFA or/and the ABCD1 gene.

**Electronic supplementary material:**

The online version of this article (10.1186/s12883-019-1449-5) contains supplementary material, which is available to authorized users.

## Background

X-linked adrenoleukodystrophy (OMIM# 300100) is an inherited disorder of peroxisomal metabolism, typically presented with impaired degradation of saturated VLCFA [1, 2]. Childhood cerebral ALD and AMN are the two most common forms of ALD, while these two phenotypes have different clinical and pathological characteristics. Generally, cerebral ALD is early onset and renders a poor prognosis, with a representative feature of rapidly progressive neuroinflammatory of cerebral demyelination. Known as a form of X-ALD, neurological symptoms of AMN, in general, occur from the ages of 20 to 50 and manifests as a chronic progressive paraparesis, accompanied with sensory and sphincter disturbances [3, 4]. It is worth noting that the neuropathology of AMN is different from the inflammatory cerebral form [5]. The fundermental neuropathological abnormality of AMN is a dying-back axonal degeneration. In this regard, it has a great impact to the cervical fasciculus gracilis and lumbar corticospinal tracts [5, 6]. Furthermore, the corticospinal tract in the posterior limbs of the internal capsule is also involved and may lead to secondary corticospinal tract degeneration in the midbrain, pons, medulla, and spinal cord [5, 7]. The peripheral nerves damage can also occur in AMN patients, but peripheral nerve involvement is often overshadowed by prominent spinal cord symptoms [8, 9]. According to whether there is any evidence of cerebral white matter lesions, AMN can further divided into ‘pure AMN’ and ‘AMN-cerebral’ forms. With a mean follow-up of 10 years, study showed that nearly 19% of patients with pure AMN developed into cerebral demyelination [10]. However, this threat can diminish significantly with age especially after 45 [11]. Due to the heterogeneity of clinical manifestations, it is difficult to diagnose AMN earlier. The clinical features of Chinese AMN patients have not been systematically evaluated until now. Therefore, the present research focuses on surveying the features of clinical, biochemical, neuroimaging and genetic information with Chinese AMN patients.

## Methods

Our group reviewed and investigated Chinese AMN patients followed at Chinese PLA General Hospital, from August 2012 to January 2018. Patients meeting the following two inclusion criteria were enrolled according to case histories: (a) Adult onset with spastic paraparesis, (b) cases with mutations of the ABCD1 gene or/and increased plasma levels of VLCFA, particularly the proportions of C24:0/C22:0 and C26:0/C22:0. With the basis of the above two standards, the study selected 6 AMN patients.

Clinical information were screened out case histories, which included age of neurological symptoms onset, family history, onset time of cognitive impairment, motor function, sensory disturbance, plantar reflex and other related signs, for instance, sexual dysfunction, urine disorder, bald and increased skin pigmentation. Plasma adrenocorticotropic hormone (ACTH) (normal range: 1.1–278.0 pmol/L) was tested in all six patients. Adrenal insufficiency was defined according to the baseline of plasma ACTH level at 08:00 am greater than 10.12 pmol/L. Additionally, we adopted electrophysiological and radiological studies methods to assess damage to the nervous system. We used standardized techniques at room temperature when conducting electrophysiological studies. Five of six patients underwent nerve conduction studies. Furthermore, four of six patients also performed somatosensory evoked potential tests. Brain and spinal MRI scans were performed in all six patients. All patients underwent 1.5 T magnetic resonance imaging, of which 2 patients underwent MRI enhanced scan. Conventional SE sequence included T1-weighted images; T2-weighted images; Diffusion-weighted images and T2-fluid-attenuated inversion-recovery sequence. Gadolinium spray was used as the enhancer, the neuroradiologist of Jing Tang reviewed all neuroimaging results. VLCFA were detected by gas chromatography - mass spectrometry in five of six patients. The ABCD1 gene mutations characterized by a custom-made target exome capture panel (MyGenostics, Beijing, China) were analyzed in five of six patients (patient 3 refused to perform genetic test for economic reasons).

## Results

### Clinical characteristics

Table 1 lists the clinical characteristics of the six AMN patients. Median age at onset of neurological symptoms was 29.8 years and ranged 21–38 years. Duration of follow-up from onset ranged between 47 months to 128 months. The mother of patient 6 with obvious skin pigmentation and we confirm her disease by gene sequencing. The other two patients (patient 2 and 4) with suspected positive family history: the maternal cousin of patient 2 had suffered from limb weakness and cutaneous pigmentation, but he refused a further check. The maternal uncle of patient 4 had symptom of ambulation dysfunction, unfortunately we could not identify his disease because of his rejection.

**Table 1:** Clinical Characteristics of 6 Chinese Patients with Adrenomyeloneuropathy

Patient number	Sex/Age (years)	Onset age (years)	Spastic paraparesis	Sensory deficit	AMN with/without cerebral involvement	Memory / mood impairment (onset age)	Adrenal dysfunction	Sphincter disturbances	Sexual dysfunction	Change of skin / hair	Family history
1	M/30	21	+	+	+	–	+	+	+	+	–
2	M/45	38	+	+	–	–	+	–	+	+	+−
3	M/44	33	+	+	–	–	+	+	+	+	–
4	M/31	26	+	–	–	–	+	+	+	+	+−
5	M/40	31	+	+	–	–	–	+	–	+	–
6	M/41	29	+	+	+	+(39)	+	+	+	+	–

“*M*” Male,”+” positive/with,“-” negative/without, “+−” suspicious

Initial neurological symptoms included stiffness or weakness of the legs in five patients and backache in one left (patient 4). Among them, two patients (patients 1, 4) were initially suspected as hereditary spastic paraplegia because of adolescent onset with spastic paraparesis, and other two patients (patients 2, 3) were initially misdiagnosed as subacute combined degeneration due to damage of dorsolateral funiculus and peripheral nerve.

**Fig. 1:**
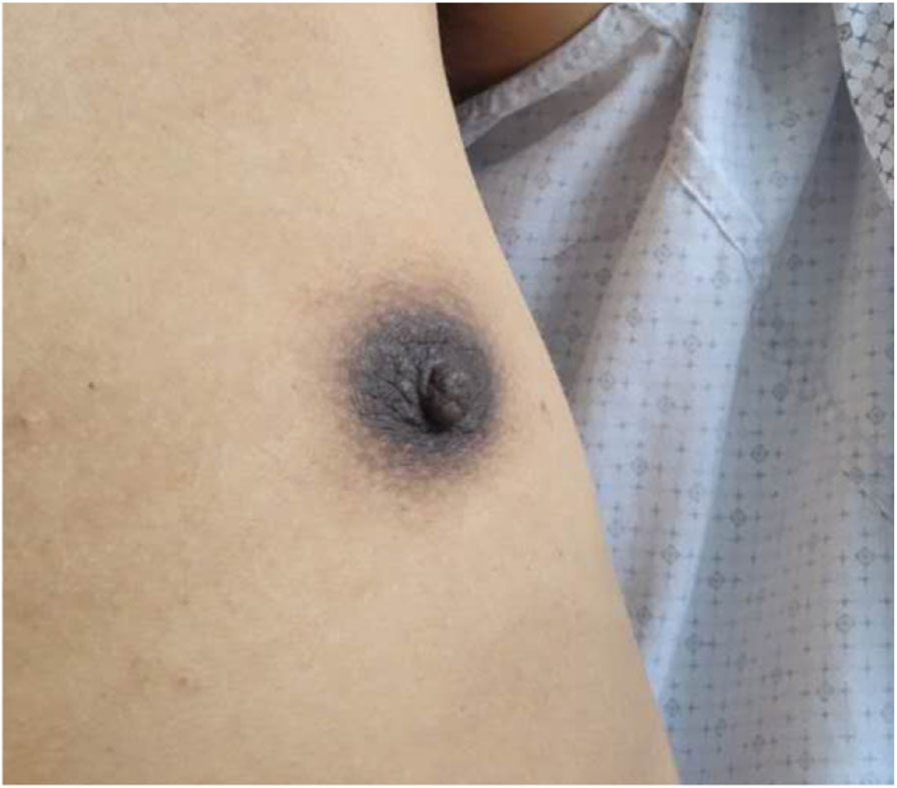
Hyperpigmentation of the body of patient 2, especially with areolae

**Fig. 2:**
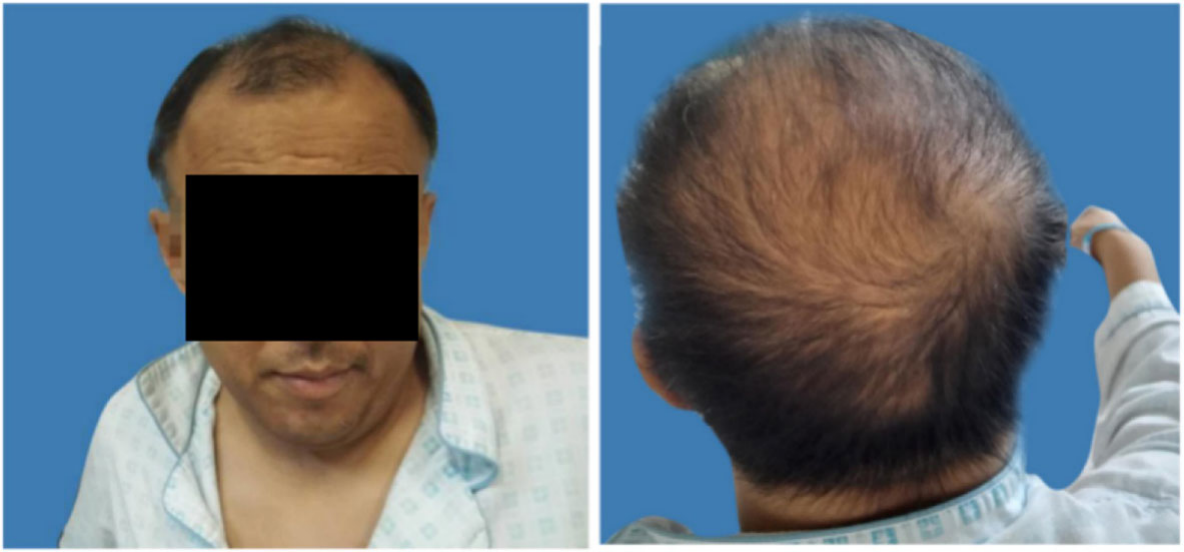
Patient 6, a 39 years old man, developed male type balding

Five patients had hyperpigmentation in lips, areolae, gums even the entire body skin (Fig. 1), and the plasma of ACTH concentration of these patients was elevated obviously. After patient 1 and patient 3 had received hydrocortisone therapy regularly, the level of plasma of ACTH returned to normal after 2–4 weeks. Moreover, the symptoms of skin pigmentation and fatigue also significantly improved. Five patients complained decreased libido and erectile dysfunction, but the levels of testosterone were in the normal range. In our cohort, all patients with scanty scalp hair, patient 6 developed male pattern baldness (Fig. 2).

On neurologic examination, all patients presented spasticity and paresis, as well as increased tendon reflexes with Babinski sign. Five patients had urinary disturbance (retention, urgency or incontinence). Patient 6 demonstrated personality change, memory impairment and dysarthria at the age of 39. Although there were lesions in the brain, patient 1 had no obvious personality change or memory impairment. Nerve conduction studies showed abnormalities in 4 of 5 tested patients. Three patients with bilateral peroneal velocity abnormal, the other one with peroneal velocity and amplitude decreased. (The detailed data about NCV can get from the Additional file 1). Somatosensory evoked potential experiments proved that 4 monitored patients had central conduction abnormalities.

### Neuroimaging

**Fig. 3:**
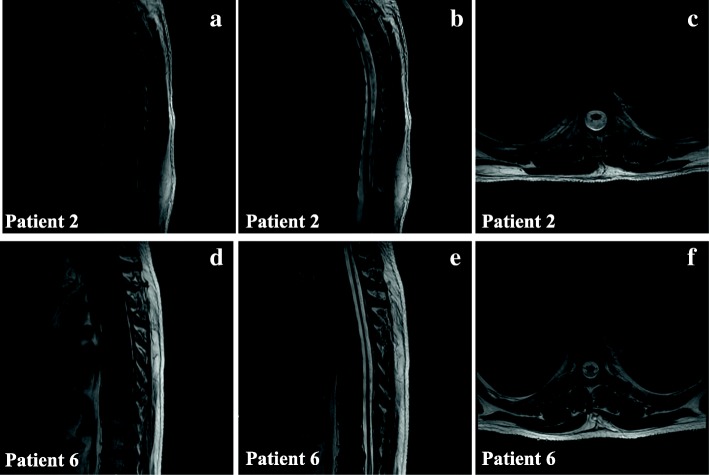
Spinal MRI scans of patient 2 and patient 6 show diffuse cord atrophy. **a** Sagittal T1-weighted images of patient 2. **b** Sagittal T2-weighted images of patient 2. **c** Axial T2-weighted images of patient 2. **d** Sagittal T1-weighted images of patient 6. **e** Sagittal T2-weighted images of patient 6. **f** Axial T2-weighted images of patient 6

**Fig. 4:**
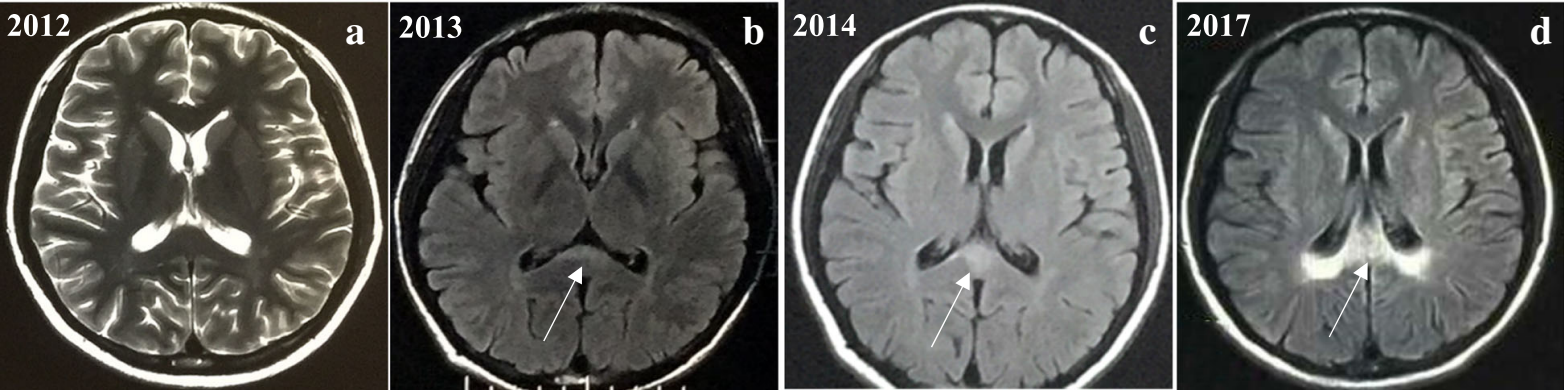
The evolution of brain MRI scans of patient 1 during last 5 years. **a** The axial T2-weighted images of brain MRI of 2012 is normal. As indicated by the arrows. **b** The axial T2-flair images of head MRI of 2013 showed a slight hyperintensity of splenium of corpus callosum. **c** The axial T2-flair images of head MRI of 2014 showed more abnormal signal of splenium of corpus than in 2013. **d** T2-flair images of head MRI of 2017 revealed marked hyperintensity of whole splenium of corpus callosum

**Fig. 5:**
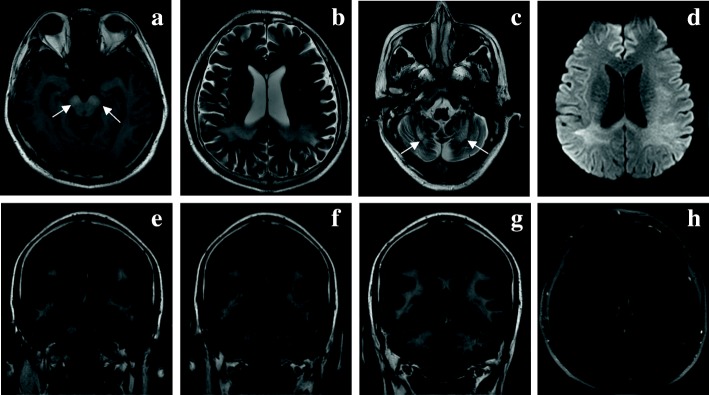
MRI of the brain images of patient 6 with AMN, shown rapidly deteriorated symptoms with cognitive decline. **a** The lesion showed increased signal intensity with bilateral corticospinal tract (arrows) on T1-weighted images. **b-c** The extensive lesions involved the parieto-occipital white matter, splenium of the corpus callosum and bilateral dentate nuclei of cerebellar (arrows) on T2-weighted images. **d** The lesion showed slightly increased signal intensity with the parieto-occipital white matter and splenium of the corpus callosum on Diffusion-weighted images. **e-g** The extensive lesions involved the bilateral temporo-parietal-occipital white matter, splenium of the corpus callosum, bilateral corticospinal tract and bilateral dentate nuclei of cerebellar on T2-flair images. **h** Axial T1‑weighted images after gadolinium administration

All patients underwent brain and spinal MRI testing. As a result, spinal MRI scans showed diffuse cord atrophy in all cases, especially patient 2 and patient 6 (Fig. 3). Interestingly, patient 1 didn’t have any obvious mood or memory disturbance until the last follow-up date, but the lesion of splenium of corpus callosum have become more and more visible during the past 6 years (Fig. 4), which indicates that the initial lesions usually relate to the splenium of the corpus callosum and subsequently spread to the adjacent white matter of the parieto-occipital lobe. The brain MRI of patient 6 revealed extensive intracranial lesions, including splenium of corpus callosum, bilateral symmetrical temporo-parietal-occipital white matter, bilateral corticospinal tract, and bilateral dentate nuclei of cerebellar (Fig. 5).

### Biochemical

Table 2 lists the plasma VLCFAs levels of five tested patients. While patient 5 refused to undergo this check after genetic diagnosis. It is revealed that plasma levels of VLCFA, and the proportions of C24:0/C22:0 and C26:0/C22:0 were elevated in all tested patients. Although patient 1 received treatment with Lorenzo’s oil regularly, the plasma levels of C26:0, especially the C24:0/C22:0 and C26:0/C22:0 ratios were still above normal range.

**Table 2:** Plasma VLCFAs levels of five Chinese patients with Adrenomyeloneuropathy

Patient	C22:0 (25.49–74.51 μmol/L)	C24:0 (20.85–61.50 μmol/L)	C26:0 (0.22–0.74 μmol/L)	C26:0/C22:0 (0.004–0.016)	C24:0/C22:0 (0.69–0.96)
1(before treated with Lorenzo’s oil)	42.5	62.3	1.12**↑**	0.09**↑**	1.63**↑**
1(after treated with Lorenzo’s oil)	10.1**↓**	14.8**↓**	0.76**↑**	0.16**↑**	1.56**↑**
2	49.1	102.4**↑**	2.81**↑**	0.057**↑**	2.09**↑**
3	40.8	66.8	1.21**↑**	0.070**↑**	2.00**↑**
4	30.2	58.4	1.48**↑**	0.049**↑**	1.93**↑**
6	38.0	70.0	2.34**↑**	0.062**↑**	1.84**↑**

*VLCFAs* very long chain fatty acids

### Mutational analysis

**Fig. 6:**
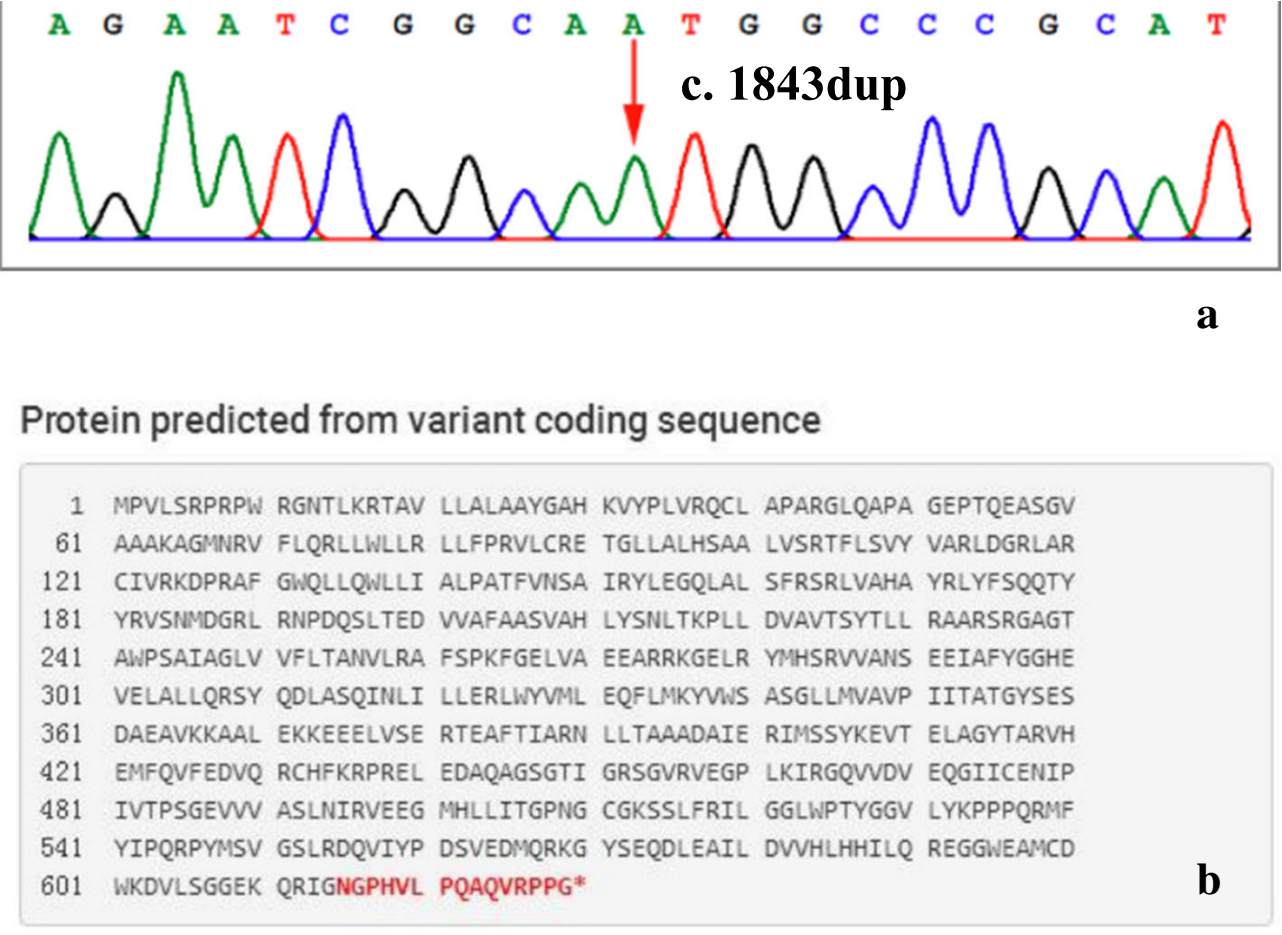
**a** The sequencing profiles of the novel mutation of patient 6. **b** The novel frameshift mutation caused the changing of the 615th amino acid located in exon 8, which leaded to the protein early terminated at exon 9

To analyze mutational features, five distinct mutations of the ABCD1 sequence were confirmed (Table 3). Among these five different mutations, four are missense, and one is a novel frameshift mutation. As to the missense counterparts, three mutations are identified in the nucleotide binding domains (NBD), and the other one is located in the transmembrane domain. The ALD database (see http://www.x-ald.nl/) actually has disclosed these 4 mutations, which are not only associated with AMN but also with the phenotype of childhood cerebral ALD, adolescent cerebral ALD, adult cerebral ALD, or the asymptomatic patients [12–15]. In this study, one novel frameshift mutation (c.1843dup) was identified in our cohort. As aforementioned, the ABCD1 gene coding for a protein of 745 amino acids (named ALDP), is formed from 10 exons [16]. Here the novel frameshift mutation locates in exon 8, which leads to the protein early terminated at exon 9 (Fig. 6).

**Table 3:** The Mutational Analysis of five Chinese patients with Adrenomyeloneuropathy

Patient number	Exon	Nucleotide change	Allele	Protein localization	Type	Reference
1	8	c.1849C > T	p. Arg617Cys	NBD	Missense	Kumar et al., 2010 [12]
2	1	c.346G > A	p. Gly116Arg	TMD	Missense	Feigenbaum et al.,1996 [13]
4	8	c.1817C > T	p. Ser606Leu	NBD	Missense	Fanen et al.,1994 [14]
5	3	c.1166G > A	p. Arg389His	TMD to NBD	Missense	Kok et al.,1995 [15]
6	8	c.1843dup	p. Met615fs^a^	NBD	Frameshift	Novel

*TMD* transmembrane domains, *NBD* nucleotide-binding domains. ^a^:termination codon

## Discussion

Even a series of progress have been accomplished on the AMN, a comprehensive exploration with perspective of clinical, neuroimaging, biochemical, and genetic analysis in Chinese AMN patients is still lack.

AMN was proposed due to the involvement of a slowly progressive paraparesis, adrenal insufficiency, hypogonadism combined with peripheral nerves neuropathy and sphincter disturbances [4]. With the discovery and application of VLCFA, AMN was soon found to be a form of phenotypic X-ALD [17, 18]. Nevertheless, neuropathologic study has revealed a more indolent degenerative process, different from the inflammatory cerebral phenotypes [5, 19]. Moreover, reports elucidated that the genotype–phenotype correlation in ALD patients is not evident [20–23]. In our cohort, although they each had the same genotype, the various clinical manifestations of patients 1, 2, 4, and 5 were consistent with the AMN phenotype, which is inconsistent with previously reported results [12–15]. These data mean that in addition to primary defect in ABCD1, environmental factors and/or other genes show a strong connection with the development of the disease. This study further confirms that genotype and phenotype may not correlate.

In our cohort, median onset age of neurological symptoms was 29.8 years and ranged 21–38 years, consisted with previous reports [24, 25]. Five of these patients developed skin pigmentation when they were young. Interestingly, patient 5 had no apparent skin color changes or abnormal plasma ACTH up to the date of follow-up. Moser et al. have suggested that there were 33% of AMN patients with normal adrenal function at all points (normal cortisol response to ACTH and normal baseline ACTH levels). Nevertheless, it’s likely that this percentage was underestimated, which may be attributed to part of neurologists who didn’t have sufficient knowledge to interpret the result as adrenal function is normal [24]. Therefore, we think that “myeloneuropathy-only” is an important phenotype of ALD. There were two of six patients (patient 1 and 6,) with cerebral involvement (33%), higher than that (about 19%) reported by van Geel et al., but lower than that (about 63%) from de Beer et al. [10, 26]. It is noteworthy that the patient’s mental state, walking ability and verbal ability showed significant deterioration about half a year after the divorce. According to previous reports, head trauma may be a possible environmental trigger [27–29] but other modifiers (both genetic and environmental) have not yet been identified. We boldly guess that emotional trauma (such as divorce) may be an enviromental factor, which intiates or aggravates cerebral disease.

MRI is an important tool for diagnosis and detecting the evolution of the disease. As displayed in Fig. 4, patient 1’s head MRI evolution indicated that intracranial lesions originate from the splenium of the corpus callosum and then increasingly extend to the periventricular and occipital-parietal white matter. Interestingly, patient 1 didn’t show any obvious mood, memory disturbance or other psychiatric symptoms, which implies that white matter lesions on MRI may preact symptoms. It is consistent with the findings of van der Knaap et al. [30, 31]. Therefore, regular head MRI evaluation is very important for patients with AMN or ALD [32, 33]. The brain MRI of patient 6 showed extensive lesions, including splenium of corpus callosum, bilateral symmetrical temporo-parietal-occipital white matter, bilateral corticospinal tract, and bilateral dentate nuclei of cerebellar, some lesions confluent. The lesions were compatible with previous results of Loes et al. [34, 35]. Although no enhancement of demyelinating lesions of patient 6’s head MRI were observed, diffusion-weighted images showed slightly increased signal intensity with the splenium of the corpus callosum as well as parieto-occipital white matter. Meanwhile, the patient experienced a considerable decline in cognitive function. It demonstrates that he develops into the rapidly progressive neuroinflammatory stage and his condition will deteriorate progressively.

Although patient 1 received Lorenzo’s oil treatment regularly, plasma C26:0 levels were nearly normal, intracranial lesions are still developing. It is compatible with previous studies [36, 37].

In our cohort, we found a novel frameshift mutations in patient 6 (Fig. 6). The Sanger sequencing results of ABCD1 gene in the family demonstrated a nucleotide insertion mutation A at cDNA nucleotide 1843(c.1843dup) which was observed in the patient’s mother. In addition, this frameshift mutation was not reported in previous studies. Combining with the clinical, imaging and biochemical characteristics of the patient, we recommend to define the mutation as Pathogenic.

## Conclusions

In conclusion, this study aims at illuminating basic features from the clinical, neuroimaging, biochemical, and genetic aspects of some Chinese AMN patients. A de novo mutation stemmed from ABCD1 gene was identified. Plasma VLCFA and ABCD1 gene analysis should be tested for the patients who present with Addison disease and spastic paraparesis, especially encountering with ‘myeloneuropathy-only’. It is also critical to diagnose AMN rapidly owing to the insistent demands of genetic counseling and applications of targeted and specific treatment options.

## Additional file

Additional file 1: The detailed data about NCV of patients. (DOCX 19 kb)

## Data Availability

All data generated or analyzed during this study are included in this published article.

## Abbreviations

ABCD1: ATP binding cassette subfamily D member 1
ACTH: Adrenocorticotropic hormone
ALD: Adrenoleukodystrophy
AMN: Adrenomyeloneuropathy
MRI: Magnetic Resonance Imaging
OMIM: Online Mendelian Inheritance in Man
PLA: Chinese People’s Liberation Army
VLCFA: Very long chain fatty acids
X-ALD: X-linked adrenoleukodystrophy
