# Supplementary material for: Clinical, neuroimaging, biochemical, and genetic features in six Chinese patients with Adrenomyeloneuropathy
Source: BMC Neurol. 2019 Sep 16;19:227. doi: 10.1186/s12883-019-1449-5 (PMC6745787; doi:10.1186/s12883-019-1449-5)
Supplement: Supplementary file 1 — The detailed data about NCV of patients. (DOCX 19 kb) [file 12883_2019_1449_MOESM1_ESM.docx]

NCS of patient 1

|  | Median(Motor) | Ulnar(Motor) | Peroneal(Motor) | Tibial(Motor) |
| --- | --- | --- | --- | --- |
| P.lat | 7.3 | 7.7 | 13.0 | 13.8 |
| P.amp | 9.7 | 15.6 | 5.6 | 8.9 |
| D.lat | 3.0 | 3.2 | 4.2 | 4.0 |
| D.amp | 9.8 | 17.6 | 6.0 | 13.4 |
| Velocity | 58.1 | 49.1 | 40.9 | 44.9 |
|  | Median(Sensory) | Ulnar(Sensory) | Sural(Sensory) |  |
| Lat | 2.5 | 2.4 | 2.8 |  |
| Amp | 7.3 | 7.4 | 11.0 |  |
| Velocity | 64.0 | 54.2 | 53.6 |  |

NCS of patient 2

|  | Median(Motor) | Ulnar(Motor) | Peroneal(Motor) | Tibial(Motor) |
| --- | --- | --- | --- | --- |
| P.lat | 8.3 | 8.7 | 16.2 | 16.5 |
| P.amp | 15.2 | 12.7 | 1.7 | 7.1 |
| D.lat | 3.8 | 3.0 | 6.0 | 4.3 |
| D.amp | 16.9 | 13.2 | 2.1 | 11.3 |
| Velocity | 53.3 | 52.6 | 32.4 | 32.8 |
|  | Median(Sensory) | Ulnar(Sensory) | Sural(Sensory) |  |
| Lat | 2.6 | 2.6 | 3.0 |  |
| Amp | 7.7 | 3.7 | 7.4 |  |
| Velocity | 61.5 | 50.0 | 50.0 |  |

NCS of patient 5

|  | Median(Motor) | Ulnar(Motor) | Peroneal(Motor) | Tibial(Motor) |
| --- | --- | --- | --- | --- |
| P.lat | 7.7 | 9.3 | 15.5 | 13.7 |
| P.amp | 10.5 | 12.8 | 3.6 | 7.2 |
| D.lat | 3.8 | 4.5 | 5.0 | 4.2 |
| D.amp | 11.7 | 12.9 | 4.6 | 7.1 |
| Velocity | 53.1 | 55.6 | 35.2 | 45.3 |
|  | Median(Sensory) | Ulnar(Sensory) | Sural(Sensory) |  |
| Lat | 4.2 | 4.3 | 2.7 |  |
| Amp | 5.2 | 4.9 | 23.0 |  |
| Velocity | 55.1 | 44.9 | 55.6 |  |

NCS of patient 6

|  | Median(Motor) | Ulnar(Motor) | Peroneal(Motor) | Tibial(Motor) |
| --- | --- | --- | --- | --- |
| P.lat | 8.8 | 8.7 | 17.0 | 18.5 |
| P.amp | 12.1 | 11.3 | 2.1 | 3.3 |
| D.lat | 4.2 | 3.2 | 6.3 | 7.0 |
| D.amp | 12.8 | 11.2 | 1.9 | 5.4 |
| Velocity | 54.3 | 57.1 | 29.9 | 36.5 |
|  | Median(Sensory) | Ulnar(Sensory) | Sural(Sensory) |  |
| Lat | 3.2 | 3.1 | 3.0 |  |
| Amp | 4.2 | 3.7 | 15.0 |  |
| Velocity | 53.1 | 46.7 | 50.0 |  |

(1)Patient 3 didn’t complete EMG.There were no abnormality with EMG in patient 4.

(2)P.lat= Proximal latency P.amp= Proximal amplitude D.lat=Distal latency D.amp= Distal amplitude

All patients underwent NCS in which their skin temperature was maintained at 32°C or above during the examination. NCS were performed on the median, ulnar, tibial, peroneal, and sural nerves using the Keypoint electromyography (EMG) system (Medoc Ltd, Israel). The results were measured according to the normal reference values utilized by the EMG laboratory of Chinese PLA General Hospital (median motor nerve: amplitude ≥5.0 mV, velocity ≥50.0 m/s; median sensory nerve: amplitude ≥5.0 µV, velocity ≥50.0 m/s; ulnar motor nerve: amplitude ≥5.0 mV, velocity ≥50.0 m/s; ulnar sensory nerve: amplitude ≥5.0 µV, velocity ≥50.0 m/s; tibial motor nerve: amplitude ≥5.0 mV, velocity ≥40.0 m/s; peroneal motor nerve: amplitude ≥3.0 mV, velocity ≥45.0 m/s; and sural sensory nerve: amplitude ≥6.0μV, velocity ≥50.0 m/s). NCS were considered abnormal if any of the studied parameters was found to be abnormal.
